# Using standardized patients for undergraduate clinical skills training in an introductory course to psychiatry

**DOI:** 10.1186/s12909-023-04107-5

**Published:** 2023-03-15

**Authors:** Jakob Siemerkus, Ana-Stela Petrescu, Laura Köchli, Klaas Enno Stephan, Helen Schmidt

**Affiliations:** 1 Translational Neuromodeling Unit (TNU), Institute for Biomedical Engineering, University of Zurich and ETH Zurich, Zurich, Switzerland; 2 Max Planck Institute for Metabolism Research, Cologne, Germany

**Keywords:** Standardized patients, Simulated patients, Undergraduate, Medical School, Psychiatry, Psychotherapy, Communication

## Abstract

**Background:**

The goal of this study was to assess the value and acceptance of Standardized or Simulated Patients (SPs) for training clinically inexperienced undergraduate medical students in psychiatric history taking, psychopathological assessment, and communication with psychiatric patients.

**Methods:**

As part of a newly developed introductory course to psychiatry, pairs of 3rd year medical students conducted psychiatric assessments of SPs, including history and psychopathological state, under the supervision of a clinical lecturer. Prior to the assessment, students attended introductory lectures to communication in psychiatry and psychopathology but were clinically inexperienced. After the interview, the students’ summary of their findings was discussed with other students and the lecturer. Students, lecturers, and actors were invited to a survey after the course. Questions for the students included self-reports about perceived learning success and authenticity of the interviews.

**Results:**

41 students, 6 actors and 8 lecturers completed the survey (response rates of 48%, 50%, and 100%, respectively). The survey results indicated that, despite their lack of clinical experience, students learned how to conduct a psychiatric interview, communicate in a non-judgmental and empathetic manner, take a psychiatric history and perform a psychopathological examination. SPs were perceived as authentic. The survey results suggested that this setting allowed for an enjoyable, non-distressful and motivating learning experience within a restricted time frame of just two afternoons.

**Conclusion:**

The results indicated that the SP approach presented is useful for teaching clinical skills in psychiatry to students with limited previous clinical experience and knowledge of psychiatry. We argue that SPs can be used to teach practical psychiatric skills already during an early phase of the curriculum. Limitations of our study include a limited sample size, a temporal gap between the course and the survey, reliance on self-reports, and lack of comparison to alternative interventions.

**Supplementary Information:**

The online version contains supplementary material available at 10.1186/s12909-023-04107-5.

## Background

Learning to work with patients is undoubtedly an integral part of training to become a physician. However, requirements of modern curricula have raised the question on how to provide students with a more standardized, controllable learning experience. One of the approaches that emerged out of this necessity was to use actors who portray symptoms as if they were patients with certain medical conditions. Since the first accounts of systematic usage of such “standardized” or “simulated” patients (SPs) dating back to the 1960s (e.g. [4]), the method has received attention in many fields of medical education.

Working with SPs builds on various concepts of adult learning. Numerous authors have motivated the didactic approach with reference to the concept of experiential learning (e.g. [2]) which follows a cycle of concrete experience, reflective observation, abstract conceptualization, and active experimentation [3]. Feedback from and discussions with clinical lecturers and other students can guide and augment the learning experience [4]. In addition, the use of SPs will typically incorporate elements of case-based learning, which is widely used in medical education and facilitates the transfer from theoretical knowledge to clinical reasoning and practice [5]. This makes the method suitable for teaching more complex clinical skills.

There are some aspects that make the assignment of SPs particularly interesting for teaching psychiatry. The method has shown its usefulness for teaching communication skills [6–9], psychopathology [10, 11] and other clinical competences that are important in psychiatric practice [12, 13]. The actors can provide feedback, both from the perspective of their role as a patient and from their professional view as actors. Ideally, this kind of structured feedback can enhance the ability of the students to reflect on their own behavior, a crucial skill in psychiatric practice [9]. Teachers can present a diversity of psychopathological phenomena to the students and even allow for exposure to acute symptoms, without endangering the patient or the students. Finally, a teaching approach with SPs is perceived as a motivating enjoyable experience [14, 15] and could help trigger interest in the field. Using SPs for teaching in psychiatry could therefore contribute to addressing the significant recruitment problems of the field [16, 17]. According to a position paper by the World Psychiatric Association, recruitment in psychiatry can be improved by including exposure to clinical psychiatry very early in the curriculum [18]. This recommendation is at odds with classical medical curricula in German-speaking countries such as Switzerland, where this study was conducted and where psychiatry is traditionally taught towards the end of the curriculum.

Our unit [19] was responsible for designing a novel introductory block course to psychiatry as part of a new Bachelor program in Human Medicine at ETH Zurich [20]. We decided to familiarize students with clinical psychiatry early in their studies and intended to train clinical skills – including communication, psychiatric history taking and psychopathology – in clinically inexperienced students. One challenge was the restricted time frame (one week) allotted to this block course. Inspired by concepts of experiential learning mentioned above, we deliberately decided to involve SPs in a practical course. Prior to the practical course, students received only limited theoretical knowledge about psychiatry in lectures (and were thus thrown in “at the deep end”). While it has been shown that the use of SPs is more effective than classical lectures for teaching communication skills of inexperienced students [8], the effectiveness for training other clinical skills in psychiatry, such as taking the psychiatric history, has received less attention or was not assessed in isolation [13]. Although the value of SPs for teaching psychopathology for inexperienced students has not been examined in direct comparison with traditional approaches such as lectures, it did show generally positive results [10, 11, 21]. Since communication, history taking and particularly psychopathology in psychiatry represent challenging topics for many students, we reasoned that interactions with SPs could prove effective to induce experiential, hands-on learning. The advantages of the approach may also outweigh previously reported limitations of SPs, for example, they can feel less authentic, and may not trigger empathy, compassion, or transference processes in the same way as real patients [22, 23].

Our course therefore included several features that differed from other progressive psychiatry curricula in German-speaking countries (e.g. [24, 25]), in particular:

i) Students received training in clinical skills in psychiatry, including psychopathological state examination, at a very early stage during their studies.
ii) Students were clinically inexperienced and, prior to their first practical exercise, had only attended introductory lectures about communication, diagnostic principles, and psychopathology.
iii) Our course relied entirely on SPs to train clinical skills.

In summary, in this study, we assessed the value and acceptance of using SPs for teaching clinical skills in psychiatry to clinically inexperienced undergraduate medical students, as part of a newly developed undergraduate psychiatry course conducted in Switzerland [19].

## Methods

### Procedure

Interaction with SPs was included in the practical section of a newly implemented compulsory introductory course to psychiatry. The target group were undergraduate 3rd year medical students who were the first cohort of a novel curriculum at ETH Zurich (Bachelor in Human Medicine) [20]. 84 students attended the practical course (44 female, 40 male; approximate mean age 22 years). Before the first practical session, students had very limited knowledge about psychiatry, but had already attended courses on general communication and history taking in medicine. Prior to the practical exercises, students attended three lectures (approximately one hour each) on communication in psychiatry, the principles of diagnosing mental illnesses, and an introduction to psychopathology and the AMDP system [26] as basis for the psychopathological examination. Prior to the practical course they were informed explicitly that they were going to interview SPs and not actual patients. Students were also provided with written preparation material, including a list of generic questions for the assessment of psychopathology. Depending on their individual schedules, some students had attended additional introductory lectures to major psychiatric disorders and typical psychopathological syndromes. The goal of this course was that students learned to communicate in a non-judgmental and empathetic fashion, and learned (from practical experience) how to take a psychiatric history and to conduct a psychopathological assessment. A secondary goal was that students learned to reflect upon their behavior, skills, and knowledge in the interaction with patients suffering from mental health problems. Students were not training for an Objective Structured Clinical Examination (OSCE).

Actors were recruited by and trained in collaboration with the Standardized Patient Program of the Learning Center at the University of Zurich. All actors were professionally trained or at least experienced actors. Clinical psychiatrists (HS, ASP, and JS) wrote four detailed scripts describing fictitious patients with major psychiatric disorders (alcohol addiction, psychosis, bipolar disorder, depression). The scripts included information about the biography, medical history (including social aspects and family history), psychopathological and somatic symptoms, but also information on how to behave in different sections of the interview or when certain issues were addressed. Care was taken to add a sufficient amount of complexity, while still scripting typical cases with moderate to severe symptoms. Each case also included elements of case-based learning without the need of external feedback or information *during* the interview, e.g., SPs were instructed to respond differently to certain types of communication, forcing students to adjust their behavior. The actors were trained in a session of 3 h by HS, ASP or JS and the Standardized Patient Program (one session with three actors for each case). In general, we followed implementation recommendations for SPs by Kühne et al. [27].

All lecturers were clinical psychologists or psychiatrists, either from our unit or from psychiatric institutions in the area of Zurich.

Students attended two afternoon sessions of 4 ½ hours each, in groups of 7–8 students. Each session started with 30 min of preparation in which the case bulletins were handed out and tasks were assigned. Pairs of two students were chosen to conduct the interviews in the role of physicians, while the lecturer and remaining students were observing. Students were informed again that they were about to interview SPs and not actual patients. Their tasks were to (i) take a history, (ii) examine the psychopathology according to the AMDP system, and (iii) summarize their findings to the lecturer and other students afterwards. Students were required to take the role as physician at least once and were encouraged to observe and reflect on their own emotions during the interview. The other students were also assigned tasks, in particular, each student was to observe one of the following aspects: (i) beginning of interview, (ii) communication technique, (iii) nonverbal communication, (iv) doctor-patient-relationship including empathy, (v) closure of interview. For students to pass the course, lecturers had to confirm that students had actively participated once as interviewers and in all other interviews as active observers, including the discussions and feedback sections.

Once roles and tasks had been assigned, the SP was welcomed and interviewed by the two students acting as physicians (60 min). After the interview was completed and the SP had left the room, both students and actors were asked to reflect on the interaction (5 min). The actors were then re-invited to the plenum and gave structured feedback on the interaction and how they felt as a SP, followed by open discussion of the students, actor and lecturer (10 min). Then psychopathological findings, history and the characteristics of the case were presented by the interviewers and student observer feedback was discussed (25 min), followed by individual feedback of the lecturer (5 min). After a break of 30 min, a second interview session took place. The afternoon was concluded by an open discussion/debriefing (30 min). See Table 1 for an overview.

**Table 1:** Breakdown of one afternoon of the practical course

Introduction	30 min	Discussion/preparation, allocation of tasks	
Two sessions	60 min	2 students’ interview with SPs; other students as observer	
	5 min	Students reflect on allocated tasks, actor transitioning and preparation of feedback for the students	
	10 min	Structured feedback from the actor and discussion	
	25 min	Cases presented by students, open discussion of findings, discussion of peer feedback	
	5 min	Individual oral feedback from lecturer for the “interviewers” (followed by 30 min break and 2nd session)	
Wrap-up	30 min	Discussion/debriefing	

Description and approximate duration of the different sections of one afternoon of the practical course.

### Ethics and survey

Students (n = 85), actors (n = 12) and lecturers (n = 8; only those lecturers not involved in designing the course and this research project) were invited four months after the course (see Discussion) via e-mail to fill out a web-based online questionnaire after giving informed consent (IC). The participation was voluntary. The questionnaire also covered other aspects of the course than the clinical exercises (e.g. the theoretical lectures) which will be reported elsewhere. We used LimeSurvey Version 3.17.7 + 190,627 for the questionnaire. Data collection took place within two weeks after invitation.

The survey was conducted in German and consisted of questions specifically designed for this course. The questionnaire served to assess potential distress of students, fulfillment of learning objectives, authenticity of interaction, and the usefulness of feedback. Furthermore, the overall student experience was assessed, including questions about preference for SPs over real patients.

Regarding the survey for the students, 23 questions concerned the practical section of the course. Lecturers were asked to answer 14 questions, and actors 13 questions. Most of the questions (students 21, lecturers 12, actors 10 questions) were designed as ordinal 4-point Likert rating scales (“disagree”, “rather disagree”, “agree”, “strongly agree” with a given statement; additional option “no answer”). Few questions required a free text input (students 2, lecturers 3, actors 4). In Table 2, selected items and associated responses are listed. In the Supplementary Table, all items and responses are listed (translated into English by the authors) and arranged with regard to the research questions (i – v).

### Data preparation and analysis

Questionnaire results were imported into IBM® SPSS® Statistics 25 for analysis.

The analysis presented in this paper uses descriptive statistics (absolute count of responses and valid percentage excluding missing values). Free text answers were not systematically analyzed, but were inspected to provide context for the interpretation of ratings.

## Results

### Participation

Of the 85 students, 41 complete datasets were obtained. All eight invited lecturers participated. Six complete data sets are available for the actors. In Table 2, a selection of responses of students is presented. For a detailed overview of responses from all participants see Supplementary Material 3. An overview of free text answers can be found in Supplementary Material 2.

### Distress

About two thirds (68%) of the students considered the task of interviewing the SP “easy”, indicating that most students did not feel overwhelmed by the task. Only seven students (17%) agreed or strongly agreed of having felt distressed by the interaction with the actors.

### Learning objectives

All of the lecturers agreed/strongly agreed that the setting of the interaction with SPs was suitable for taking the medical history and for examining the psychopathology – and that the students had been able to use the interview for these purposes. The majority of the students stated that they were able to take the medical history (90%) and to examine the psychopathological state (78%). Furthermore, 83% of the students reported that they had had a neutral/non-judgmental attitude towards the SP which was confirmed by the lecturers’ responses (100% strongly agreed) and the actors’ responses (five agreed, one strongly agreed). Only one lecturer did not agree that the setting of the interaction with the SP was suitable for identifying typical problems in the interaction with patients. 90% of the students reported that they agreed or strongly agreed that the practical course improved their competency in talking to patients. Also, being a student observer during the interviews was rated as beneficial for improving one’s competency in interactions with patients (88% agreement or strong agreement).

### Authenticity

The vast majority of students stated that the interaction with the SP felt real (51% agreed and 29% strongly agreed; see Table 2) and that the interactions triggered empathy/compassion (44% agreement, 34% strong agreement). The lecturers strongly agreed that the cases were designed realistically/authentically (88%) and agreed (37.5%) or strongly agreed (62.5%) that the cases were realistically/authentically presented by SPs. All lecturers strongly agreed that students adopted an empathetic attitude towards the SPs.

### Direct feedback

Students indicated that problems arising during the interviews and the reflection upon these problems were helpful to improve their skills and knowledge (37% agreed, 61% strongly agreed). The vast majority of students also perceived the debriefings with actors as helpful to reflect on their own behavior (39% agreed, 56% strongly agreed). The debriefing section with the lecturer was perceived as helpful in improving knowledge and communication skills (two thirds of the students strongly agreed). Each of the lecturers agreed/strongly agreed that the special setting of the course made it possible to use mistakes or incompleteness of the interviews for further discussion and improvement of the learning success.

### Overall experience and preferences of the participants

The majority of students either agreed (46%) or strongly agreed (20%) that they would have found it harder to apply their knowledge and skills in a setting with a “real” patient. Only 22% of the students indicated that they would have preferred to conduct the interview with real patients, while more than two thirds of the students disagreed or strongly disagreed with this alternative. Almost all students (98%) and all lecturers agreed or strongly agreed with the use of actors in this course. Additionally, all actors indicated that, based on their experience, they would recommend an engagement as an actor in this course. Notably, more than two thirds of students indicated that the practical course had triggered their interest in the field of psychiatry (41% agreed, 37% strongly agreed). The course was perceived as lively and enjoyable by the students (22% agreed, 73% strongly agreed).

**Table 2:** Responses to selected items (students)

	disagree	rather disagree	agree	strongly agree	no answer
The discussions with the “patient” (actor) have burdened me emotionally.	37%	41%	12%	5%	5%
The conversations with the “patient” (actor) felt like a real situation in my role as a doctor.	0%	12%	51%	29%	7%
Possible problems in the interview situation and their reflection in the debriefing helped me to deepen my knowledge and improve my skills for medical consultations.	0%	0%	37%	61%	2%
The practical exercises make me feel more competent to talk to “real” patients with psychiatric problems.	0%	5%	44%	46%	5%
The debriefings with the actor have helped me to reflect on my own behavior.	0%	0%	39%	56%	5%
The debriefings with the lecturer helped me to deepen my knowledge and improve my conversational skills for a role as medical doctor.	0%	0%	27%	68%	5%
I think I would have found it harder to apply my knowledge and skills to a “real” patient.	2%	27%	46%	20%	5%
I would have preferred to have had the interviews with “real” patients.	15%	54%	12%	10%	10%
I would generally recommend the use of actors in this course.	0%	0%	32%	66%	2%
Through the practical exercises I have developed an interest in psychiatry.	2%	10%	41%	37%	10%

Percentage of responses to selected items (41 students). Rounded to full percentages. For a table with complete responses (frequencies and percentages) for all participants see the Supplementary Material 3. Table 1

## Discussion

In this study we used a web-based questionnaire to evaluate the usefulness of SPs during training of clinical skills in a psychiatry course for clinically inexperienced undergraduate medical students with limited knowledge of psychiatry.

Prior to the course, an open question was whether the practical exercises would be accessible to students with limited experience in communication with patients and history taking and with practically no knowledge about psychopathology. During the preparation of the course, several lecturers expressed concerns that the students might be overwhelmed by “being thrown in at the deep end”, i.e., being asked to conduct a one-hour clinical interview. On the contrary, our results clearly indicate that students did not feel overwhelmed by the interview session. Most of them indicated that they found the interaction with the SP “easy” and that they enjoyed the course. Only a few students expressed that they had felt distress as a consequence of the interview. In the free text responses, a very limited number of students pointed out that they felt stressed because of limited knowledge and skills before encountering the first interview, but not because of the interaction with the SP per se. The majority of students indicated that they preferred SPs over the interaction with an actual patient. This could either mean that students liked the interaction with a SP more or did not feel confident enough to interact with a real patient. We conclude that, in our setting, the interaction with the SPs enabled a non-stressful and enjoyable learning experience for the students.

Even though we did not include a structured exam, the students’, lecturers’ and actors’ responses similarly indicated that the learning objectives were met: Students were communicating non-judgmentally, were empathetic, were able to explore the history of the cases and assess the most important aspects of the psychopathology. The opportunity to learn in a structured setting, the open and anxiety-free environment, the discussions in the course and the active observation of the students in the practical session appear to have contributed to these encouraging results.

In many courses with SPs, feedback is given in breaks between shorter interview segments so that students can apply the insights from external feedback [cf. 24]. We deliberately chose a longer interview, comparable to a clinical consultation, in order to enhance the authenticity of the experience. An authentic, experiential, “hands-on” learning experience can translate even limited theoretical knowledge into clinical reasoning and practical skills that prepare students for their clinical rotations. A disadvantage of our approach was, however, that professional, SP and peer feedback [4] was only provided after the interview. Students therefore could not directly use the feedback to change their behavior in the interviews.

Taken together, our results show that students can learn meaningful clinical skills without much previous knowledge. The generally positive findings are in line with previous evidence on learning success in courses that use SPs in psychiatric training for medical students [8, 9, 15, 24, 28, 29].

We believe that interactions with SPs can improve communication skills and help to develop professional behavior, most importantly, because it inspires self-reflection of the medical students, a skill that is not always fostered sufficiently by traditional medical curricula. As one of the central skills in handling difficult situations with patients with mental disorders, it is very encouraging that students indicated that they were able to reflect on their behavior based on the feedback from the actors. Feedback from the clinical professional, as well as peers [4], further enhanced this learning experience.

The clinical lecturers were satisfied with the authenticity of the cases and the presentation by the SPs. This underlines that the investment in scripting multi-faceted cases and careful training of the SPs, as well as collaborating with a professional partner with long-standing experience in training SPs for medical education paid off. Also, the case histories and the psychopathological profiles were deliberately scripted to resemble typical but complex cases, distinct from the short descriptions of patients in textbooks, in order to stimulate student engagement and enhance the learning experience as suggested by Brenner [30] or Wuendrich et al. [31]. It was encouraging to see that students did not express discontent about the authenticity or “realness” of the interactions with the SPs either, a finding that we attribute to the professionalism of the actors, the in-depth training and the supervision of the actors within the Standardized Patient Program. Our findings that students reported that they were emotionally touched by the interaction with the SPs, that they felt empathetic towards the SPs, and their commitment in the interview are somewhat in contrast to the study by Krahn et al. [23]. Our approach therefore created emotional involvement while maintaining a “playful” atmosphere which has been shown to enhance learning in medical students [32]. Another point contradicting previous work [23, 33] was that students reported to prefer SPs over actual patients. Since most students had only limited experience with actual patients, reservations and also anxiety to confront an actual patient (regardless of the illness) could have contributed to this tendency. Nevertheless, at least in the context of this course, with clinically inexperienced medical students, concerns about the authenticity of the experience with SPs are unwarranted.

Our positive findings are underlined by the fact that the vast majority of students reported that they did not only find the exercises lively and enjoyable but also that the practical course triggered their interest in the field of psychiatry. Furthermore, students reported that, after the course, they felt more competent to deal with interactions with actual patients. These findings may also be attributed to the SP approach used [14, 15]. Inspiring enthusiasm in students is of crucial importance to the field of psychiatry [17] that struggles with recruitment problems globally [16].

Compared to previously published studies, our work is novel in several respects. In our study, SPs were professionals or at least very experienced actors while many previous studies either used psychiatric staff to portray cases or did not explain how they recruited and trained SPs (e.g. [11, 15, 28, 29, 34]). Furthermore, we employed SPs to train students in psychiatry clinical skills beyond communication, including psychiatric history taking and assessment of psychopathology. In our study, we assess the value of SPs for training, an approach which is only rarely investigated in studies [8, 13, 15, 24, 28, 29], when compared with the number of studies on SPs use in OSCEs (e.g. [34–37]).

### Limitations

The participation rate for students and actors was limited (48% of students, 50% of actors). Besides the fact that participation was not mandatory nor integral part of the curriculum, the informed consent and the formal character of the survey probably represented a hurdle for many participants [38]. Despite these circumstances, our response rates do not compare poorly to the response rate in voluntary course evaluations of medical schools [39] and a relevant non-response bias in course evaluation in medical education seems unlikely [39, 40].

A major limitation of this study is its descriptive and retrospective character. The participants were invited only after the course had been conducted and therefore ratings were retrospective. Moreover, due to the disruptions caused by the COVID-19 pandemic, with the first Swiss lockdown happening shortly after the course, there was a considerable gap between the end of the course and the invitation of the participants (four months). While it is unclear whether the timing of course evaluations could lead to more positive or negative ratings [cf. 41, 42], the generally positive findings could indicate a selection bias for participants who had a very positive experience in the course.

With respect to possible distress of students we did not use sophisticated questionnaires. To draw more informed conclusions, the *Therapist Response Questionnaire* [43] could be used in future studies as it was designed to cover countertransference and would therefore encourage students to reflect on their emotions during the interviews.

This study did not include a control condition that we could compare the results to. Interviews with both SPs and real patients, embedded in a crossover study design, would be ideal to gain insight in the pros and cons of both teaching approaches. There was no structured exam either which could have provided a more objective measure of learning success or efficacy of the training, respectively. Finally, the tightly timed schedule in this block course did not allow us to implement an OSCE that is suited to assess practical skills.

## Conclusion

Based on our findings, we would conclude with the following practice points for the implementation of SP programs:

- SPs are a useful method for teaching clinical skills in psychiatry, even for students who are clinically inexperienced and have limited theoretical knowledge. The method can be used early during undergraduate studies and can be combined with more advanced clinical skills training such as assessments of psychopathology.
- Cases should be typical but at the same time complex enough to provide an authentic experience for the students. Scripts for the actors should include case-based learning elements.
- We would recommend working with professional actors who have been trained by clinical professionals.
- Adequate time for feedback and discussion should be included to allow for student self-reflection and to augment experiential learning.

Despite its small size and retrospective nature, our study illustrates how new teaching formats focusing on clinical skills and exploiting the strengths of SPs can evoke very positive reactions by medical students. In line with previous reports (e.g. [44]), we hope that such positive experiences may change the students’ attitudes towards psychiatry as a discipline and increase their willingness to consider a clinical career in psychiatry.

## Electronic supplementary material

Below is the link to the electronic supplementary material.

Supplementary Material 1. Information

Supplementary Material 2. Material

Supplementary Material 3. Table 1

## Data Availability

Tables containing frequencies of all responses from the survey are included in this published article and in Supplementary Material 3. The dataset used in this article is available from the corresponding author on reasonable request. An example for a case description (in German) can be requested from the corresponding author.

## List of abbreviations

OSCE: Objective Structured Clinical Examination
SP: Standardized Patient, Simulated Patient
