# Supplementary material for: Using standardized patients for undergraduate clinical skills training in an introductory course to psychiatry
Source: BMC Med Educ. 2023 Mar 15;23:159. doi: 10.1186/s12909-023-04107-5 (PMC10016160; doi:10.1186/s12909-023-04107-5)
Supplement: Supplementary file 1 — Supplementary Material 1. Information [file 12909_2023_4107_MOESM1_ESM.docx]

***Supplementary Material 1***

Information on free text answers can be found in Supplementary Material 2. Material.

A table containing frequencies and percentages of all responses (students, lecturers and actors) from the survey with relevance to this article is included in Supplementary Material 3. Table 1.
