# Supplementary material for: Using standardized patients for undergraduate clinical skills training in an introductory course to psychiatry
Source: BMC Med Educ. 2023 Mar 15;23:159. doi: 10.1186/s12909-023-04107-5 (PMC10016160; doi:10.1186/s12909-023-04107-5)
Supplement: Supplementary file 2 — Supplementary Material 2. Material [file 12909_2023_4107_MOESM2_ESM.pdf]

Supplementary Material 2. Material

Using Standardized Patients for Undergraduate Clinical Skills Training in an Introductory Course to Psychiatry

Jakob Siemerkus[1], Ana-Stela Petrescu[1], Laura Köchli[1], Klaas Enno Stephan[1,2], Helen Schmidt[1]

[1] Translational Neuromodeling Unit (TNU), Institute for Biomedical Engineering, University of Zurich and ETH Zurich, Zurich, Switzerland

[2] Max Planck Institute for Metabolism Research, Cologne, Germany

Corresponding Author: Jakob Siemerkus

Email Address: siemerkus@biomed.ee.ethz.ch

Free Text Answers

A total of 22 students provided free text responses to the question “What aspects did you like in the practical courses?”. Many students stated that they liked the interaction with the SPs and described the debriefing as being helpful for their learning experience. Some students expressed their acknowledgment of the actors’ performance. A few students also explained that they appreciated the “non-stressful” environment for their first practical learning experience in psychiatry. A small number of students reported that they had felt distressed because they had to conduct the interview with only limited knowledge about the disorders. Some lecturers reported that they liked the performance of the SPs and working with them.
