# Supplementary material for: Using standardized patients for undergraduate clinical skills training in an introductory course to psychiatry
Source: BMC Med Educ. 2023 Mar 15;23:159. doi: 10.1186/s12909-023-04107-5 (PMC10016160; doi:10.1186/s12909-023-04107-5)
Supplement: Supplementary file 3 — Supplementary Material 3. Table 1 [file 12909_2023_4107_MOESM3_ESM.pdf]

Supplementary Material 3. Table 1

Using Standardized Patients for Undergraduate Clinical Skills Training in an Introductory Course to Psychiatry

BMC Medical Education

Jakob Siemerkus[1], Ana-Stela Petrescu[1], Laura Köchli[1], Klaas Enno Stephan[1,2], Helen Schmidt[1]

[1] Translational Neuromodeling Unit (TNU), Institute for Biomedical Engineering, University of Zurich and ETH Zurich, Zurich, Switzerland

[2] Max Planck Institute for Metabolism Research, Cologne, Germany

Corresponding Author: Jakob Siemerkus

Email Address: siemerkus@biomed.ee.ethz.ch

Supplementary Material 3. Table 1: Responses from students, lecturers and actors

Responses from students, lecturers and actors										
**i) Distress**										
	disagree		rather disagree		agree		strongly agree		no answer	
	n	%	n	%	n	%	n	%	n	%
**Students**										
The interview with the "patient" was easy for me in my role as a doctor (procedure and conducting the interview).	0	0%	10	24%	21	51%	7	17%	3	7%
The discussions with the "patient" (actor) have burdened me emotionally.	15	37%	17	41%	5	12%	2	5%	2	5%
**Actors**										
Playing the patient role in my profession as an actor was easy for me in the interview with the students.	0	0%	0	0%	4	67%	2	33%	0	0%
Preparing and playing the patient role was similar or less stressful compared to my regular work as an actor.	0	0%	1	17%	5	83%	0	0%	0	0%
The conversations with the students have burdened me emotionally in my role as a patient.	4	67%	1	17%	1	17%	0	0%	0	0%

ii) Learning objectives										
	disagree		rather disagree		agree		strongly agree		no answer	
	n	%	n	%	n	%	n	%	n	%
**Students**										
The AMDP documents (sample questions and short description of the items for the AMDP psychopathology scale) were helpful in the practical exercises.	0	0%	3	7%	14	34%	24	59%	0	0%
The prior information on the cases in the practical exercises was helpful to get engaged in the interview.	0	0%	5	12%	17	41%	15	37%	4	10%
The schedule for the course afternoons and the time allocated to the different sections was appropriate.	0	0%	6	15%	11	27%	22	54%	2	5%
The prior discussions with the lecturer were helpful.	0	0%	6	15%	16	39%	16	39%	3	7%
I have been neutral towards the “patient” (actor) and have not judged them.	0	0%	3	7%	18	44%	16	39%	4	10%
I was able to take the history.	1	2%	1	2%	25	61%	12	29%	2	5%
I was able to examine the mental (psychopathological) state.	0	0%	4	10%	23	56%	9	22%	5	12%
Through the role as an observer I have been able to improve my own skills for medical interviews.	0	0%	3	7%	22	54%	14	34%	2	5%
The practical exercises make me feel more competent to talk to “real” patients with psychiatric problems.	0	0%	2	5%	18	44%	19	46%	2	5%
**Lecturers**										
I found the students well prepared and motivated.	0	0%	0	0%	1	13%	7	88%	0	0%
The setting of the simulated interviews was suitable for taking a medical history.	0	0%	0	0%	3	38%	5	63%	0	0%
The setting of the simulated interviews was suitable for a psychopathological examination.	0	0%	0	0%	3	38%	5	63%	0	0%
The setting of the simulated interviews was suitable for identifying typical difficulties in the assessment of patients in psychiatry	0	0%	1	13%	3	38%	4	50%	0	0%
The students were able to collect the most important anamnestic information during the interviews and were able to describe it afterwards.	0	0%	0	0%	5	63%	3	38%	0	0%
The students were able to identify the central psychopathological characteristics in the interviews and were able to describe these afterwards.	0	0%	0	0%	4	50%	4	50%	0	0%
The students behaved in a non-judgmental or neutral manner towards the simulated patients.	0	0%	0	0%	0	0%	8	100 %	0	0%
**Actors**										
I felt understood by the students in my role as a patient.	0	0%	0	0%	4	67%	1	17%	1	17%
The students did not judge me in my role as a patient.	0	0%	0	0%	5	83%	1	17%	0	0%

iii) Authenticity										
	disagree		rather disagree		agree		strongly agree		no answer	
	n	%	n	%	n	%	n	%	n	%
**Students**										
The conversations with the "patient" (actor) felt like a real situation in my role as a doctor.	0	0%	5	12%	21	51%	12	29%	3	7%
The descriptions and the behavior of the "patient" (actor) have triggered compassion (empathy) in me.	2	5%	6	15%	18	44%	14	34%	1	2%
**Lecturers**										
The cases were designed realistically.	0	0%	0	0%	1	13%	7	88%	0	0%
The cases were realistically presented by the actors.	0	0%	0	0%	3	38%	5	63%	0	0%
The students have adopted an empathetic attitude towards the "patients".	0	0%	0	0%	0	0%	8	100 %	0	0%

iv) Direct feedback										
	disagree		rather disagree		agree		strongly agree		no answer	
	n	%	n	%	n	%	n	%	n	%
**Students**										
Possible problems in the interview situation and their reflection in the debriefing helped me to deepen my knowledge and improve my skills for medical consultations.	0	0%	0	0%	15	37%	25	61%	1	2%
The debriefings with the actor have helped me to reflect on my own behavior.	0	0%	0	0%	16	39%	23	56%	2	5%
The debriefings with the lecturer helped me to deepen my knowledge and improve my conversational skills for a role as a medical doctor.	0	0%	0	0%	11	27%	28	68%	2	5%
**Lecturers**										
Even if individual aspects were incorrectly ascertained, incorrectly assessed or not asked about at all in the interviews, this was helpful for the discussion and learning success.	0	0%	0	0%	3	38%	5	63%	0	0%

v) Overall experience and preferences of the participants										
	disagree		rather disagree		agree		strongly agree		no answer	
	n	%	n	%	n	%	n	%	n	%
**Students**										
I think I would have found it harder to apply my knowledge and skills to a "real" patient.	1	2%	11	27%	19	46%	8	20%	2	5%
I would have preferred to have had the interviews with "real" patients.	6	15%	22	54%	5	12%	4	10%	4	10%
I would generally recommend the use of actors in this course.	0	0%	0	0%	13	32%	27	66%	1	2%
Through the practical exercises I have developed an interest in psychiatry.	1	2%	4	10%	17	41%	15	37%	4	10%
The practical exercises were lively and I enjoyed them.	0	0%	0	0%	9	22%	30	73%	2	5%
**Lecturers**										
I would generally recommend that the course be conducted with actors.	0	0%	0	0%	2	25%	6	75%	0	0%
**Actors**										
I would recommend an engagement as an actor because of the structure of the content preparation, the training and the interviews in this course.	0	0%	0	0%	2	33%	4	67%	0	0%

Responses (frequencies and percentages) from students, lecturers and actors arranged with regard to the corresponding main research questions (i – v). Rounded to full percentages.
